# Contrasted Genetic Diversity, Relevance of Climate and Host Plants, and Comments on the Taxonomic Problems of the Genus *Picoa* (Pyronemataceae, Pezizales)

**DOI:** 10.1371/journal.pone.0138513

**Published:** 2015-09-21

**Authors:** Fatima El-Houaria Zitouni-Haouar, Pablo Alvarado, Imed Sbissi, Abdellatif Boudabous, Zohra Fortas, Gabriel Moreno, José Luis Manjón, Maher Gtari

**Affiliations:** 1 Laboratoire de Biologie des Microorganismes et de Biotechnologie, Département de Biotechnologie, Faculté des Sciences de la nature et de la vie, Université d’Oran 1, Ahmed Ben Bella, Algérie; 2 Departamento Ciencias de la Vida, Universidad de Alcalá, 28871, Alcalá de Henares, Madrid, Spain; 3 Laboratoire d’Ecologie Pastorale, Institut des Régions Arides, 4119, Médenine. Tunisia; 4 Laboratoire Microorganismes and Biomolécules Actives, Université Tunis El Manar & Université de Carthage, Campus Universitaire, 2092, Tunis, Tunisia; Field Museum of Natural History, UNITED STATES

## Abstract

The species concept within the genus *Picoa* Vittad. is here revisited in light of new molecular and ecological data obtained from samples collected throughout the Mediterranean basin. Two highly diverse widespread clades and four additional minor lineages were significantly supported by three genes dataset (ITS, 28s LSU and RPB2) inferences for 70 specimens. The two widespread clades occur in very different geographical and ecological areas associated with exclusive host plants in the genus *Helianthemum*. SEM study of spore surface morphology in these lineages revealed the existence of smooth ascospores in the majority of these clades. However the most frequent lineage in Europe and coastal North Africa displayed either smooth or verrucose spores. Hence this morphological criterion cannot be reliably used to discriminate between the different clades. In addition, SEM observations made on ascospores from several original collections of *P*. *juniperi* and *P*. *lefebvrei* supported the hypothesis that ornamentation depends on the degree of maturity in some of these lineages. Geographical and ecological, rather than morphological data are here suggested as the most useful characters to separate the different lineages in *Picoa*. Further studies focusing on these features are needed before the names *P*. *juniperi* and *P*. *lefebvrei* can be unambiguously linked with the genetic lineages observed.

## Introduction

Specimens of *Picoa* Vittad. produce hypogeous ascomata that can be found in semi-arid and desert ecosystems of most countries around the Mediterranean basin and the Middle East [1,2,3,4,5,6,7,8,9]. They establish mycorrhizal associations with several annual and perennial xerophytic host plants in the Cistaceae, especially those in the genus *Helianthemum* [10]. These plants and their associated fungi may play a major role in the maintenance of Mediterranean shrublands and grasslands and help prevent erosion and desertification [11].

The genus *Picoa* was proposed by Vittadini [12] for *Picoa juniperi* Vittad., a hypogeous ascomycete collected in northern Italy close to *Juniperus* sp. and characterized by its black flat-warted surface, white gleba filled with pseudoveins, and globose asci with smooth globose spores. Since their first description to the present time, species of the genus *Picoa* have been subject to important changes in their nomenclatural and taxonomic position, being reassigned into other genera or else being invested as new genera themselves. In 1894, Patouillard [13] described the genus *Phaeangium* Pat. and its only species *Phaeangium lefebvrei* Pat. to accommodate a sample collected at Ras-el-Oued (southern Tunisia). He described it as a hypogeous ascomycete characterized by brownish ascoma with hairy unwarted peridium, homogeneously white gleba, and globose to subglobose stipitate asci containing smooth ovoid spores. On the basis of observations made on Hennings specimens, Maire [14] disputed the Patouillard description related to sporocarp surface and gleba structure and recombined the species as *Picoa lefebvrei* (Pat.) Maire stating the absence of clear distinctive characteristics between *Phaeangium* and *Picoa* Alsheikh & Trappe [15] reexamined the original material of *Phaeangium lefebvrei* from different localities, Tunisia, Algeria, Lybia, Iraq as well as new collections of this species from Kuwait. The spore ornamentation at maturity and the tomentous peridium placed *Phaeangium lefebvrei* again as the unique species of the *Phaeangium* genus [15]. These authors considered the smooth spores observed in the type specimen of *P*. *lefebvrei* as immature, and synonymised it with the Algerian species *Terfezia schweinfurthii* Hennings, which was reported to have warty spores. Moreno et al. [1] argued that *P*. *juniperi* and *P*. *lefebvrei* should be considered congeneric based on their similarities in peridial tomentum, overall colour of the ascoma, and spore ornamentation. Læssøe & Hansen [16] indicated that some earlier unpublished molecular results of O’Donnell et al. [17] already linked *P*. *juniperi* with the genus *Otidea* (Pers.) Bonord. within the Pyronemataceae. The first published sequences by Gutierrez et al. [10] confirmed this taxonomic affiliation. Sbissi et al. [4] agreed with the membership of both species in the genus *Picoa* close to *Geopora cooperi* Hark. within Pyronemataceae. In this work, smooth-spored samples from Tunisia were identified as *P*. *juniperi*, and those from Europe with minutely warted spores were named *P*. *lefebvrei*. The status of a third lineage of African samples with smooth spores could not be resolved. In the works of Ammarellou et al. [5] and Jamali & Banihashemi [7,8], new samples from Iran were sequenced, leading to similar conclusions. Unfortunately, no European samples with smooth spores were included in their analysis. Tedersoo and Smith [18] confirmed one more time the nesting of *Picoa lefebvrei* within *Pyronemataceae* next to its neighbor species *Geopora cooperi*. A third species in *Picoa*, *P*. *carthusiana* Tul & C. Tul, was found to be closely related to the Morchellaceae–Helvellaceae on the basis of morphological [19] and molecular data [17]. This species was combined in the genus *Leucangium* Quél. by Saccardo [20] as *Leucangium carthusianum* (Tul. & C. Tul.) Paol. It is currently considered as synonym of the type species of its genus, *Leucangium ophtalmosporum* Quél [16]. In 1956, Lange described *Picoa pachyascus* M. Lange, the first American representative species of this genus. However, it has been synonymised with the type species of *Imaia* Trappe & Kovács, *Imaia gigantea* (S. Imai) Trappe & Kovács [21]. Finally, Moreno et al. [22] described the last species in the genus, *Picoa melospora* G. Moreno, J. Díez & Manjón, which was treated later as *Tuber melosporum* (G. Moreno, J. Díez & Manjón) P. Alvarado, G. Moreno, J.L. Manjón & Díez [23].

In the present work, samples from most Mediterranean countries were analyzed in order to improve our understanding of the biogeographic and phylogenetic relationships between the different lineages of *Picoa*. SEM was employed to study spore ornamentation in newly collected specimens and original herbarium collections of *P*. *juniperi* and *P*. *lefebvrei*, and results were compared with phylogenetic data from multilocus sequences to evaluate the taxonomic importance of this morphological feature.

## Material And Methods

### Ethics statement

Truffles of the genus *Picoa* are not listed in any national or regional law as protected or endangered species. The collection of specimens was not subjected to any restriction or specific permissions. Samples were harvested from open lands that are not privately-owned or protected and no specific permissions were required for these collection sites by the local authorities.

### Fungal specimens

Ascomata were collected from a wide variety of habitats and regions across the Mediterranean basin (S1 Text), including semi-arid environments in the European shore (France, Italy, Spain, Greece), semi-arid habitats in the African shore (Algeria) and Middle East (Iran), and more deserted regions near Saharan desert in Africa (Algeria, Tunisia) and Middle East Syrian desert (Israel). Dried samples from Europe, Iran and Israel were preserved at Universidad de Alcalá herbarium (AH). Original collections of *Picoa juniperi*, *P*. *lefebvrei* and *Terfezia schweinfurthii* were studied and compared with the newly collected samples. Autoptic material from their original authors was kindly loaned by Farlow Herbarium (FH), Botanische Staatssammlung München (M), and Swedish Museum of Natural History (S). Morphological study of asci and ascospores was conducted using an Olympus CX22 microscope. Ascospore ornamentation was examined and photographed with a scanning electron microscopy (SEM) device JEOL JSM-6610LV at University of Science and Technology of Oran, or else with a Zeiss DSM-950 instrument at University of Alcalá.

### DNA extraction, PCR amplification and sequencing

DNA from Algerian specimens was extracted from approximately 25 mg of dried samples from each sample. Tissues of the gleba were ground in liquid nitrogen and the ascomata Genomic DNAs were isolated using ABIOpure^TM^ Genomic DNA Plant Extraction Kit (Alliance Bio, USA) according to the manufacturer’s instructions. Extracts were eluted in 50 μl of Elution Buffer supplied in the kit and stored at -20°C. DNA concentration was estimated using a NanoDrop spectrophotometer (Thermo Scientific). Four different loci were amplified from DNA template by means of polymerase chain reaction (PCR). ITS rDNA was amplified using the primer pair ITS1 and ITS4 [24], large ribosomal subunit (28S nLSU) was obtained using primers LR0R and LR5 [25], β-tubulin gene was amplified using the couple (Bt2a–Bt2b) [26], and RNA polymerase II second largest subunit using bRPB2-6F and bRPB2-7R [27]. PCR amplifications were performed in a 25 μl final volume. Cycling conditions consisted in an initial denaturation step at 95°C for 2 min, followed by 35 cycles of a 1-min denaturation at 94°C, annealing at 53°C (ITS rDNA) or), or 58°C (β-tubulin), 47°C (LSU rDNA) and 55°C (RPB2) for 1 min, and elongation at 72°C for 1 min, with a final extension step at 72°C for 10 min. Amplification products were analyzed in 1.5% agarose gel in 0.5× TBE buffer (89 mmol l^-1^ Tris, 89 mmol l^-1^ borate, 2 mmol l^-1^ EDTA), stained with ethidium bromide, and visualized under UV light. The PCR products were enzymatically purified by exonuclease and alkaline phosphatase (Exo/SAP) and then bi-directionally sequenced. Sequence reactions were performed using the ABI PRISM^TM^ 3130 Genetic Analyzer with Big Dye Terminator v3.1 Cycle Sequencing kit (Applied Biosystems; HTDS, Tunisia) according to the manufacturer instructions. DNA from European, Iranian and Israelite samples was extracted and amplified following the methods described in previous publications [28]. ITS1 / ITS4; LR0R / LR5; Bt2a / Bt2b and bRPB2-6F / bRPB2-7R were employed for amplification and sequencing purposes. Sequences produced are available in public databases (Table 1).

**Table 1:** Collection of *Picoa* species studied in the present work.

Taxon	Coll. N°	Origin	ITS	28S LSU	RPB2	β-tubulin
*Picoa* sp.	AH19561	Córdoba, Spain	JN392155	-	-	JN392136
*Picoa* sp.	AH37801	Zaragoza, Spain	JN392169	JN392200	-	JN392135
*Picoa* sp.	AH37802	Ciudad-Real, Spain	JN392176	JN392192	-	JN392139
*Picoa* sp.	AH38893	Guadalajara, Madrid, Spain	JN392175	JN392193	-	JN392140
*Picoa* sp.	AH38906	Burgos, Spain	JN392166	JN392198	-	JN392125
*Picoa* sp.	AH38931	Burgos, Spain	JN392153	-	-	JN392137
*Picoa* sp.	AH38956	Guadalajara, Madrid, Spain	JN392165	-	-	JN392127
*Picoa* sp.	AH39001	Oristano, Sardinia, Italy	JN392173	-	-	-
*Picoa* sp.	AH39204	Botsvuara, Israel	JN392147	JN392187	-	-
*Picoa* sp.	AH39205	Bouches-du-Rhône, Marseille, France	JN392162	JN392184	-	-
*Picoa* sp.	AH39206	Oristano, Sardinia, Italy	JN392172	JN392190	-	JN392138
*Picoa* sp.	AH39207	Oristano, Sardinia, Italy	JN392164	JN392178	-	-
*Picoa* sp.	AH39035	Albacete, Spain	JN392150	-	-	JN392141
*Picoa* sp.	AH39139	Guadalajara, Madrid, Spain	JN392149	JN392191	-	JN392142
*Picoa* sp.	AH39246	Burgos, Spain	JN392151	JN392201	KT350965	JN392134
*Picoa* sp.	AH39247	Burgos, Spain	JN392154	JN392186	KT350971	JN392131
*Picoa* sp.	AH39248	Burgos, Spain	JN392158	JN392196	-	-
*Picoa* sp.	AH39268	Guadalajara, Madrid, Spain	JN392160	JN392194	KT350978	JN392128
*Picoa* sp.	AH39269	Madrid, Spain	JN392174	JN392189	KT350976	-
*Picoa* sp.	AH39270	Guadalajara, Madrid, Spain	JN392161	JN392195	-	-
*Picoa* sp.	AH39282	Oristano, Sardinia, Italy	JN392171	JN392179	-	-
*Picoa* sp.	AH39285	L’Aquila, Italy	JN392152	JN392185	-	JN392132
*Picoa* sp.	AH39286	Fars, Iran	JN392157	JN392180	-	-
*Picoa* sp.	AH39287	Fars, Iran	JN392148	JN392181	-	JN392143
*Picoa* sp.	AH19584	Botsuvha, Israel	JN392146	JN392188	-	JN392130
*Picoa* sp.	AH37794	Madrid, Spain	JN392170	-	-	JN392129
*Picoa* sp.	AH37802	Ciudad-Real, Spain	JN392176	JN392192	KT350977	JN392139
*Picoa* sp.	AH38913	Madrid, Spain	JN392167	JN392199	-	JN392126
*Picoa* sp.	AH38914	Zaragoza, Spain	JN392168	JN392197	-	-
*Picoa* sp.	AH39204	Botsvuara, Israel	JN392147	JN392187	-	-
*Picoa* sp.	BMBH1	Tiaret, Benhamed, Algeria	KR073969	-	-	-
*Picoa* sp.	BMBH2	Tiaret, Benhamed, Algeria	KR073955	-	-	KR073935
*Picoa* sp.	BMBH3	Tiaret, Benhamed, Algeria	KR073970	-	-	KR073947
*Picoa* sp.	BMBH4	Tiaret, Benhamed, Algeria	KR073971	-	-	KR073948
*Picoa* sp.	BMBH5	Tiaret, Benhamed, Algeria	-	-	-	KR073936
*Picoa* sp.	BMBH6	Tiaret, Benhamed, Algeria	-	-	-	KR073949
*Picoa* sp.	BMBH7	Tiaret, Benhamed, Algeria	KR073956	KT350959	KT350972	KR073937
*Picoa* sp.	BMBH8	Tiaret, Benhamed, Algeria	KR073957	-	-	KR073938
*Picoa* sp.	BMBH9	Tiaret, Benhamed, Algeria	KR073972	-	-	-
*Picoa* sp.	BMBC10	Tiaret, Bouchouat, Algeria	KR073950	-	-	KR073929
*Picoa* sp.	BMBC11	Tiaret, Bouchouat, Algeria	KR073965	-	-	KR073943
*Picoa* sp.	BMBC12	Tiaret, Bouchouat, Algeria	KT350949	-	-	-
*Picoa* sp.	BMBC13	Tiaret, Bouchouat, Algeria	KR073973	KT350961	KT350974	KR073944
*Picoa* sp.	BMBC14	Tiaret, Bouchouat, Algeria	KR073974	KT350960	KT350973	KR073945
*Picoa* sp.	BMBC15	Tiaret, Bouchouat, Algeria	KR073951	-	-	KR073930
*Picoa* sp.	BMBC16	Tiaret, Bouchouat, Algeria	-	-	-	KR073946
*Picoa* sp.	BMBC17	Tiaret, Bouchouat, Algeria	-	-	-	KR073931
*Picoa* sp.	BMBC31	Tiaret, Bouchouat, Algeria	KT350950	KT350954	KT350964	-
*Picoa sp*.	BMBZ32	Tiaret, Sidi Bou Zebboudj, Algeria	KT350946	-	-	-
*Picoa* sp.	BMBD33	El-Bayadh, Mesbah, Algeria	KT350947	KT350953	-	-
*Picoa* sp.	BMBD34	El-Bayadh, Mesbah, Algeria	KT350948	-	-	-
*Picoa* sp.	BMBO18	Bechar, Beni Ounif, Algeria	KR073966	KT350956	KT350967	-
*Picoa* sp.	BMBO19	Bechar, Beni Ounif, Algeria	KR073952	KT350955	KT350966	KR073940
*Picoa* sp.	BMBO20	Bechar, Beni Ounif, Algeria	KR073953	-	-	KR073939
*Picoa* sp.	BMBO21	Bechar, Beni Ounif, Algeria	KR073958	-	-	KR073932
*Picoa* sp.	BMBO22	Bechar, Beni Ounif, Algeria	KR073959	-	-	-
*Picoa* sp.	BMBO23	Bechar, Beni Ounif, Algeria	KR073960	-	-	KR073933
*Picoa* sp.	BMBO24	Bechar, Beni Ounif, Algeria	KR073961	KT350957	KT350968	KR073934
*Picoa* sp.	BMBT25	Bechar, Tabelbala, Algeria	KR073967	-	-	-
*Picoa* sp.	BMBT26	Bechar, Tabelbala, Algeria	KR073968	-	-	-
*Picoa* sp.	BMBT27	Bechar, Tabelbala, Algeria	KR073954	-	-	-
*Picoa* sp.	BMBT28	Bechar, Tabelbala, Algeria	KR073962	-	-	KR073941
*Picoa* sp.	BMBT29	Bechar, Tabelbala, Algeria	KR073963	-	-	-
*Picoa* sp.	BMBT30	Bechar, Tabelbala, Algeria	KR073964	KT350958	KT350969	KR073942
*Picoa* sp.	IRA-MBA SBa	Medenine, Tunisia	KT350943	KT350951	KT350962	-
*Picoa* sp.	IRA-MBA SBb	Sbitla, Tunisia	KT350944	KT350952	KT350963	-
*Picoa* sp.	IRA-MBA SBc	Mahdia, Tunisia	KT350945	-	-	-
*Picoa* sp.	VK2106	Attica, Greece	JN392156	JN392177	KT350970	JN392133
*Picoa* sp.	VK2148	Attica, Greece	JN392159	JN392182	KT350975	JN392123
*Picoa* sp.	VK2043	Attica, Greece	JN392163	JN392183	-	JN392124

### Phylogenetic analysis

ITS rDNA and LSU nucleotide sequences were first compared with public databases using the BLAST algorithm [29] and then aligned with the sequences retrieved using the ClustalW application [30]. Simultaneously, a manual correction of sequences was conducted. The reference *Picoa* sequences came from Bidartondo & Doring (unpublished), Gutiérrez et al. [10], Sbissi et al. [4], and Jamali & Banihashemi [7,8]. Aligned loci were independently subjected to MrModeltest 2.3 [31] in PAUP* 4.0b10 [32]. The best models (SYM for ITS and RPB2, GTR for LSU) were implemented in MrBayes 3.1 [33], where a Bayesian analysis was performed (two simultaneous runs, six chains, temperature set to 0.2, sampling every 100th generation) until standard deviation of split frequencies was <0.01 after 1 320 000 (ITS) and 310 000 generations (LSU-RPB2-ITS). Finally a full search for the best-scoring maximum likelihood tree was performed in RAxML [34] using rapid bootstrap algorithm and model GTRMIX. Significance thresholds were set above 70% for bootstrap (BP) and 95% for posterior probability (PP).

## Results

### Phylogenetic analysis

**Fig 1:**
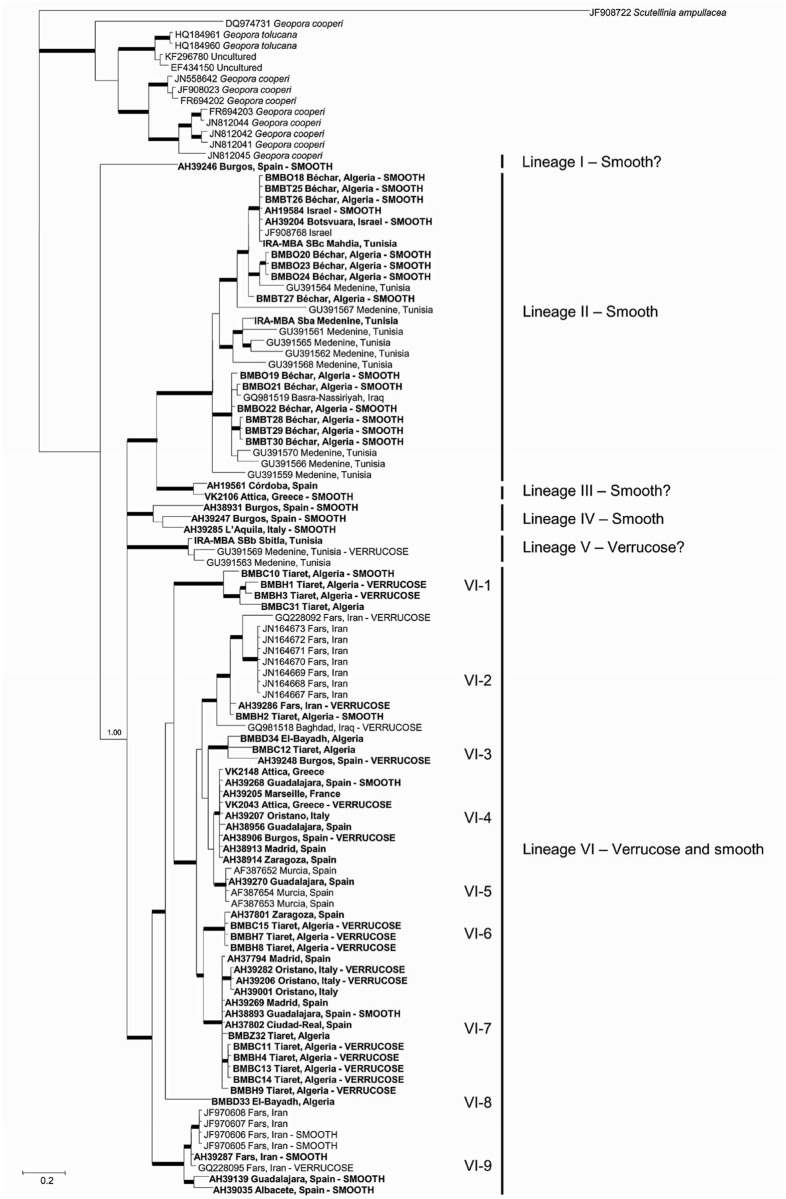
ITS consensus Bayesian phylogram of genus *Picoa* and its sister taxon *Geopora* reconstructed in MrBayes 3.1. Bold nodes are significantly supported by both inference methods employed (>70% BP and >95% PP). Nodes annotated were significantly supported by only one of these methods. Values represent Bayesian posterior probabilities, and RAxML bootstrap proportions, respectively.

**Fig 2:**
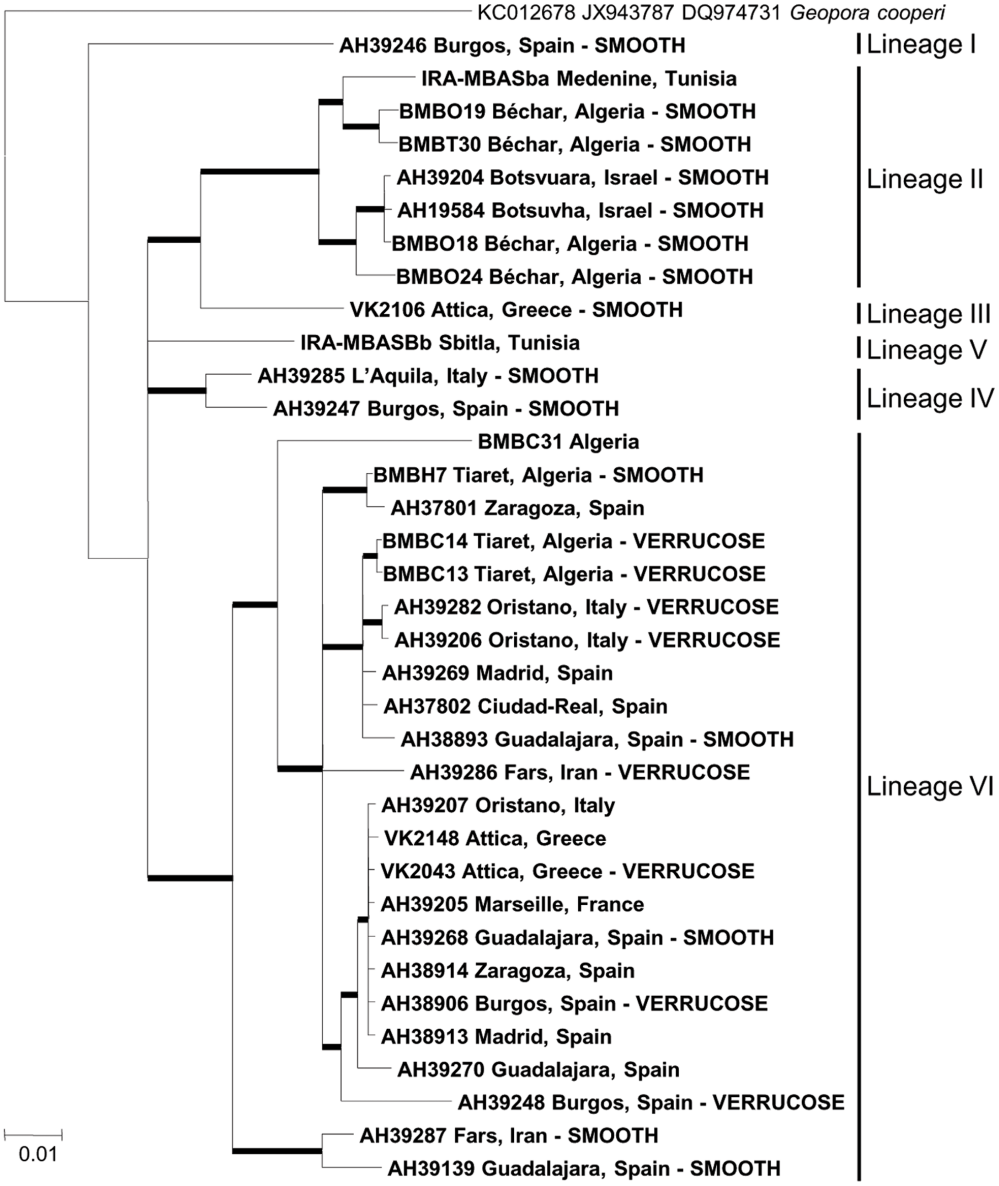
LSU-RPB2-ITS consensus Bayesian phylogram of genus *Picoa* reconstructed in MrBayes 3.1. Bold nodes are significantly supported by both inference methods employed (>70% BP and >95% PP). Nodes annotated were significantly supported by only one of these methods. Values represent Bayesian posterior probabilities, and RAxML bootstrap proportions, respectively.

The whole genus *Picoa*, with 82 new sequences, presented an overall 40.9% ITS divergence (208/508 differences, n = 86), and six different lineages were supported by phylogenetic inference. Sample AH 39246 from Spain (Lineage I) produced the most divergent sequence, being consistently identified as the most basal branch of the genus by all analyses. The remaining main clade included at least 5 major lineages (Fig 1). Lineage II is composed of exclusively African (Algeria, Tunisia) and Middle Eastern (Iraq, Israel) specimens. Lineage III is formed by the southernmost European specimens (Greece, and southern Spain). Lineage IV is composed also of European samples (Italy, Spain). Lineage V contains exclusively Tunisian specimens. Finally, the inclusive lineage VI comprises a large group of several monophyletic clades from mixed origins: VI-1 (Algeria), VI-2 (Algeria, Iran, Iraq), VI-3 (Algeria, Spain), VI-4 (France, Greece, Italy, Spain), VI-5 (Spain), VI-6 (Algeria, Spain), VI-7 (Algeria, Italy, Spain), VI-8 (Algeria) and VI-9 (Iran, Spain). Intra-lineage variability was also high: lineage II (19.2%, n = 28), lineage III (2.3%, n = 2), lineage IV (7.1%, n = 3), lineage V (3.2%, n = 3), and lineage VI (28.2%, n = 57). Within Lineage VI, the measured intra-clade variability was: VI-1 (5.6%, n = 4), VI-2 (8.2%, n = 11), VI-3 (4.4%, n = 3), VI-4 (0.2%, n = 9), VI-5 (0.4%, n = 4), VI-6 (1.3%, n = 4), VI-7 (1.1%, n = 13), VI-9 (3.7%, n = 8). On the other hand, the combined LSU-RPB2-ITS analysis successfully supported the same lineages (Fig 2). β-tubulin sequences showed scarce variability, and the analysis of this marker did not support any phylogenetic structure within *Picoa* (data not shown).

### Morphologic analysis

**Fig 3:**
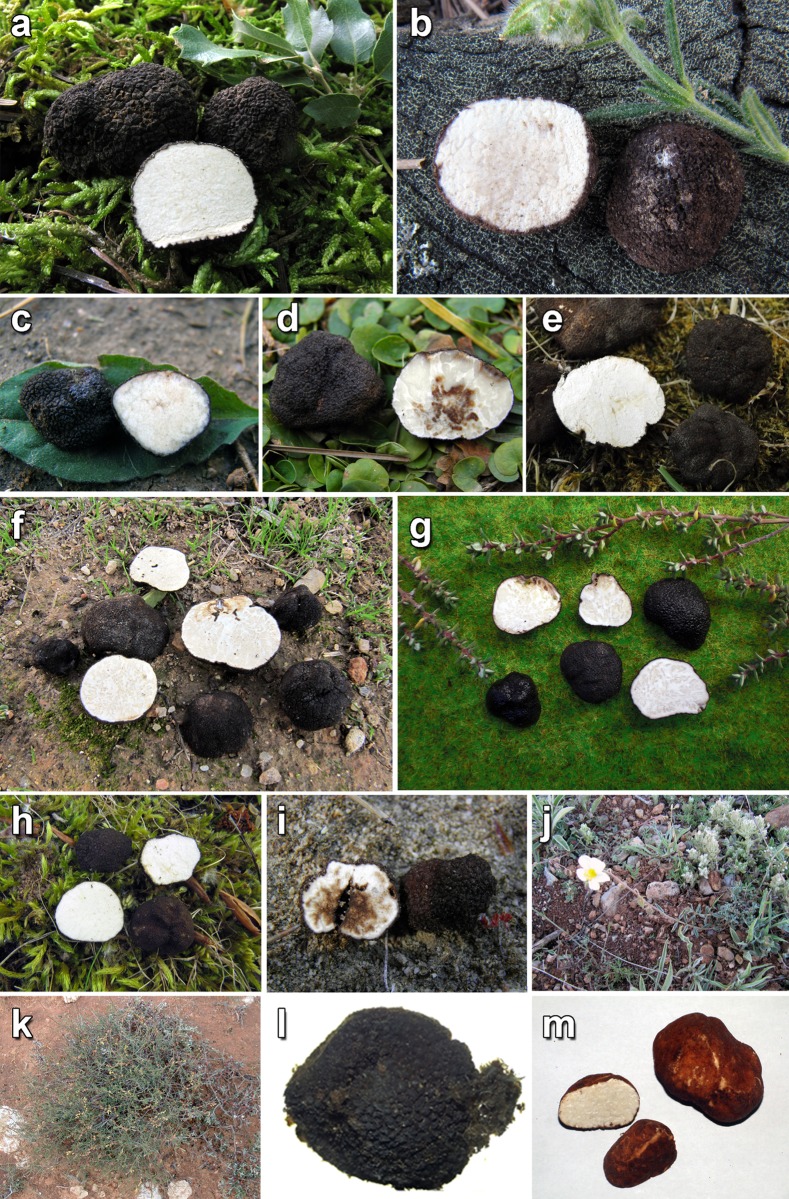
Macroscopical images of some of the samples studied. **a**. AH 39247; **b**. AH 39268; **c**. VK 2043; **d**. VK 2106; **e**. AH 38906; **f**. AH 39139; **g**. AH 38893; **h**. AH 38956; **i**. VK 2148; **j**. habitat of *Picoa* in Castilblanco de Henares (Guadalajara, Spain); **k**. habitat of *Picoa* under *Helianthemum lippii* var. *sissiliflorum*; **l**. BMBC15; **m**.BMBH4.

**Fig 4:**
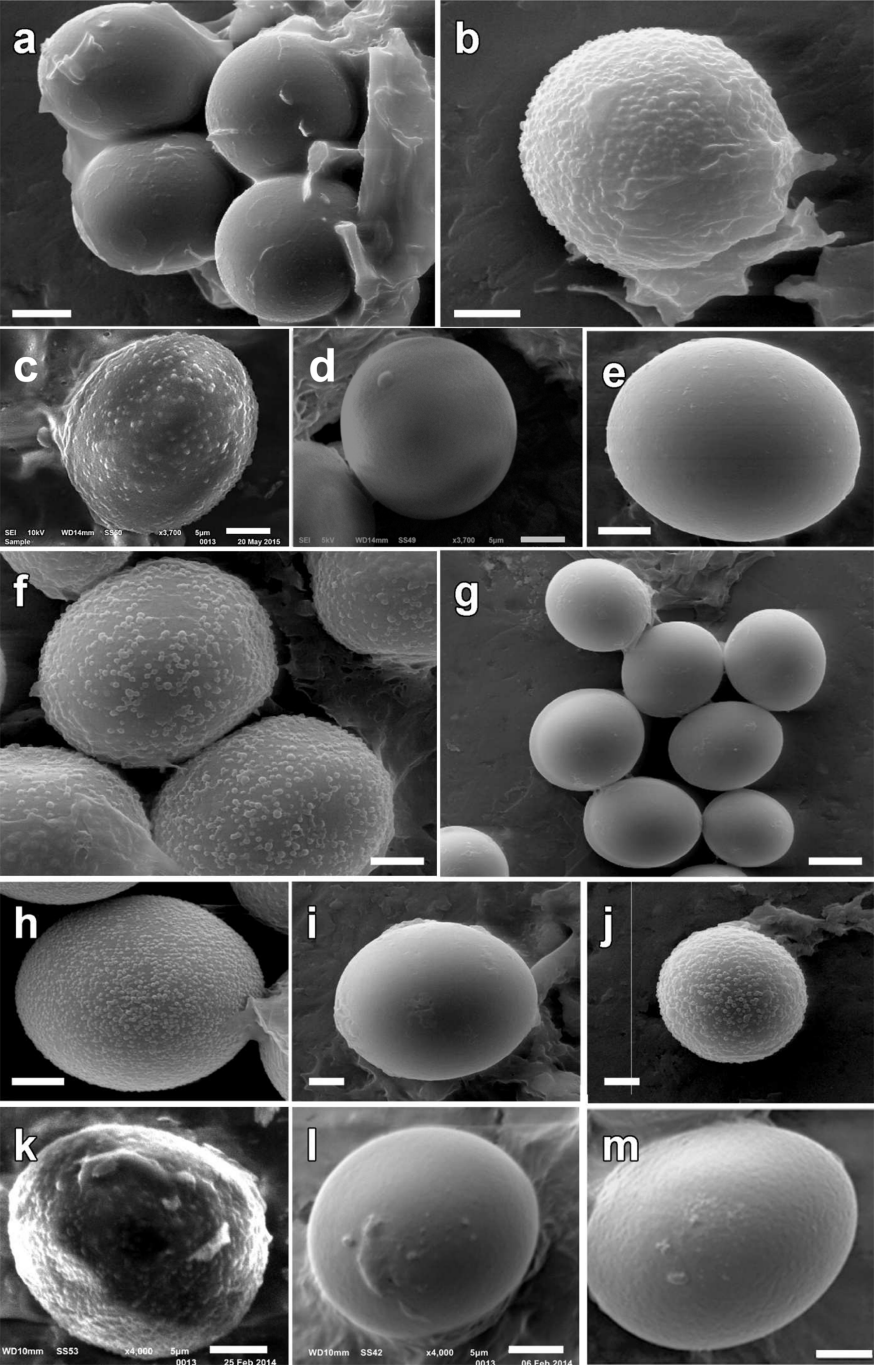
SEM and LM images of some of the samples studied. **a-b**. Autoptic specimen of *Picoa juniperi* from Vittadini’s herbarium WU10-145 ex PAD; **c**. BMBH1; **d**. BMBT26; **e**. AH 19584; **f**. M-0157945; **g**. AH 39246; **h**. AH 39286; **i**. AH 38931; **j**. AH 39206; **k**. BMBH9; **l**. BMBH5; **m**. BMBO19. Bars: **a** = 10 μm; **b** = 5 μm; **c-d** = 5 μm; **e-f** = 5 μm; **g** = 10 μm; **h-j** = 5 μm; **k-l-m** = 5 μm.

**Fig 5:**
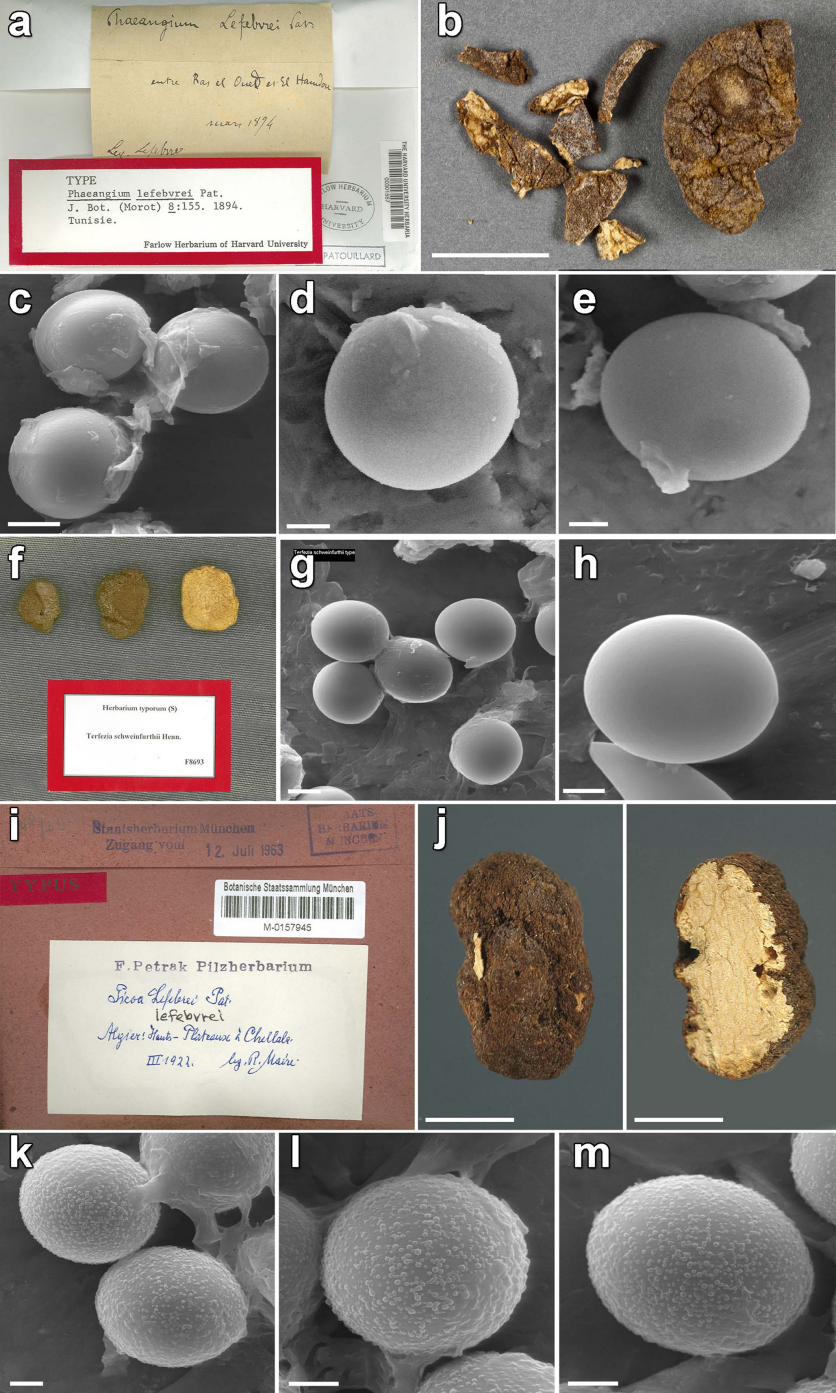
Type studies in African *Picoa* lineage. **a-e**. *Phaeangium lefebvrei* holotype FH 301557; **f-h**. *Terfezia schweinfurthii* syntype S F8693; **i-m**. *Picoa lefebvrei* M 157945. Bars: 5 μm.

Macroscopically, samples in lineages I, III and IV were black or blackish, with regularly polygonal or pyramidal warts (Fig 3). European samples in Lineage VI were black, blackish, dark grey or dark brown in color, and were covered with regularly polygonal or pyramidal warts, although some specimens were also minutely warted, giving a papillose appearance. African samples of Lineage VI were dark brown to reddish-brown, nearly smooth or covered with more or less small rounded to angular warts. African and Middle Eastern samples of lineage II were either black with pyramidal warts, brown or dark brown with minutely papillose warts, or sometimes presented widely separated warts, looking yellowish in their interspaces. Spore ornamentation was hardly visible with a light microscope and boundaries among these samples were only perceivable in SEM (Fig 4). Lineage II produces exclusively smooth spores, while Lineage VI produces mostly verrucose, but also smooth spores. All other minor clades, except the Lineage V, have smooth spores. The study of original material (Figs 4 and 5) revealed that the type collection of *Phaeangium lefebvrei* FH 301557 (leg. Lefebvre, entre Ras-el-Oued et El Hamdon, Tunisia, 1894) has perfectly smooth spores under SEM, while Patouillard’s collection Lloyd 48192 (Gafsa, Tunisia, 1898) and Maire’s collection M 157945 (Algiers, Hauts-Plateaux à Chellala, 1922) presented verrucose spores. Schweinfurth’s *Terfezia schweinfurthii* syntype S F8693 (Algeria, pr. Biskra, 1901) presented also smooth spores under SEM. The study of autoptic material of Vittadini’s *P*. *juniperi* in Mattirolo’s herbarium at Padova WU 10–145 (ex PAD) revealed the presence of both smooth and verrucose spores in the same sample.

## Discussion

**Fig 6:**
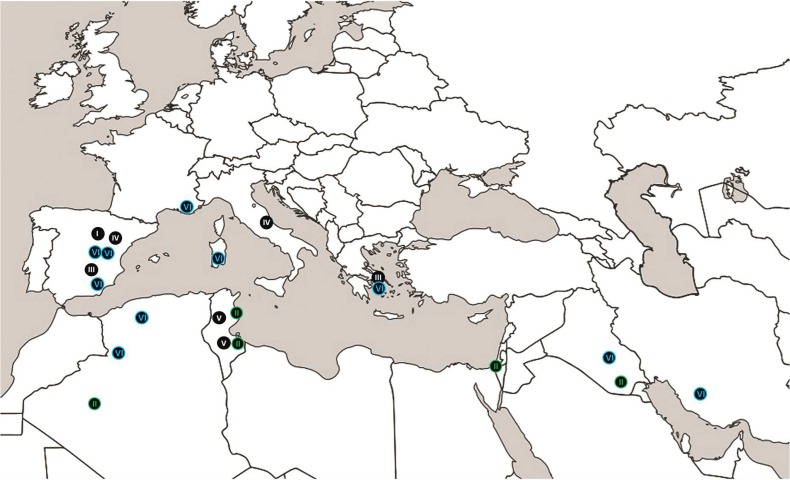
Geographic origin of the collections belonging to the two major lineages observed. Lineage II in green; lineage VI in blue.

The present work provides a comprehensive view of phylogenetic diversity in *Picoa* around the Mediterranean basin. A high degree of genetic variability was found in the whole genus and within its major clades with ITS analysis. A combined LSU-RPB2-ITS multigenic analysis largely supported the same clades obtained from ITS inference, while β-tubulin failed to support any distinct lineage at all. Among the four regions analyzed, the internal transcribed spacer (ITS) region has a high amplification and sequencing success rate, and therefore we propose ITS as the suitable barcode for *Picoa*. β-tubulin was consistently the worst performing marker, with a non-discrimination power. The ITS region has the highest probability of successful identification for the broadest range of fungi and it was formally proposed to the Consortium for the Barcode of Life for adoption as the primary fungal barcode marker [35]. A high cryptic diversity can be observed also in the sister genus *Geopora*, where a number of distinct genetic lineages with high intraspecific diversity and few characteristic morphological features exist [36], but only one of them, *Geopora tolucana*, has been proposed as a new independent taxon because of its brownish hymenium, different from the typical whitish one of *G*. *cooperi* [37]. Results from the present analyses again provide strong evidence on the close relationships between *Picoa* and *Geopora* species, particularly *Geopora cooperi;* and clearly supported the nesting of *Picoa* within the Pyronemataceae. Several studies supported the deep nesting of *Picoa* within the *Geopora* lineage [4,5,7,8,18,37,38] as a sister taxon of *G*. *cooperi* [4,18,37,38]. Perry et al. [39] pointed out a difficulty in delineating Pyronemataceae family due to the absence of clear combinations of characters, either macro- or microscopically. These authors suggest that morphological characters traditionally used to segregate this family into subfamilial groups are not phylogenetically informative above the genus level [39]. Of the six genetic lineages identified in *Picoa*, four of them (I, III, IV and V) are yet too poorly represented to draw reliable hypothesis on their ecology and distribution. The remaining two (II and VI) clearly differ in their bioclimatic origin (arid / Lineage II; semi-arid / Lineage VI) (Fig 6), and putative host plants, but share most morphological features, excepting that Lineage II never produces verrucose spores. Morphological species delimitation within *Picoa* has always been challenging since diagnostic characters have been considered often ambiguous [13,22]. Spore surface is widely thought to be smooth in *P*. *juniperi* and warty in *P*. *lefebvrei* [40], despite the fact that *P*. *lefebvrei* was originally described with smooth spores [13]. Alsheikh & Trappe (1983) [15] checked Patouillard’s type collection (FH 301557, leg. Lefebvre, entre Ras-el-Oued et El Hamdon, Tunisia, 1894) and verified it has smooth spores under light microscope, but considered them to be immature. Pacioni & El-Kholy [41] and Moreno et al. [22] found new African and Middle-Eastern collections with smooth spores under SEM, but they also attributed this fact to insufficient maturity. In the present work, we confirm that the type specimens of *P*. *lefebvrei* and *T*. *schweinfurthii* have smooth spores under SEM, in accordance with their respective protologues, while other African collections from Patouillard and Maire exhibit verrucose spores. It is here demonstrated also that the most widespread lineage in Africa and Middle East produces exclusively smooth spores. On the other hand, SEM images of an original collection of Vittadini in Mattirolo’s herbarium WU 10–145 (ex PAD) revealed the presence of smooth and verrucose spores in the same sample. This could be the result of a contamination, but most probably an intermediate developmental stage. Most newly collected European specimens of *Picoa* display exclusively verrucose spores (Lineage VI), although some collections have smooth spores instead. The same can be seen in Algerian specimens of Lineage VI. Results of these analyses reveal a strong taxonomic affinity between the two species. Whereas the lineage II was consistently smooth, the lack of spore ornamentation in immature ascomata of the remaining lineages was very likely the main difficulty that has prevailed in *Picoa* species delimitation. The genetic pattern within *Picoa* seems to correlate well with ecological features and putative host plants in the genus *Helianthemum*, which may have played a key role in their evolution. The Lineages II and V reflect a predominant presence of the perennial *Helianthemum* shrubs *H*. *lippii* in the Algerian and Tunisian specimens. This *Helianthemum* species is also reported as a putative host in Israeli desertic areas [42] although the sample AH 19584 (Lineage II) was collected under *H*. *kahiricum*. The only putative sequence from Botswana stored in GenBank is the result of an inappropriate transcription, and comes from Kibbutz Revivim, Israel (Giusto Giovanetti, pers. comm.). It is currently a part of A. Montecchi’s personal collection [40]. Lineage VI is associated with several annuals and perennials *Helianthemum* species; *H*. *salicifolium* and *H*. *hirtum* in the Algerian samples from semiarid habitats; *H*. *ledifolium* in the Iranian semiarid areas of Fars province [7,8]; *H*. *ledifolium* and *H*. *salicifolium* in the Spanish sample AH 37802 and *Juniperus oxycedrus*, *Cistus* sp., *Ephedra* sp. and *Pistacia* sp. in Italian sample AH 39001. In Spain, Honrubia [11] and Gutiérrez et al. [10] noted a mycorrhizal association between *Picoa* (Lineage VI) and *H*. *almeriense*. In France, it was linked to *H*. *nummularium* [43,44]. However, the ecological data of Lineages III and IV do not include any *Helianthemum* species. Indeed, sample VK 2106 (Lineage III) was collected under *Fumana* sp., *Pinus* sp. and *Cistus monspeliensis*; AH 38931 and AH 39247 (Lineage IV) were harvested under *Quercus* spp., with some *Cistus* plants present, Finally, sample AH 39246 (Lineage I) was found in a treeless mixed field with *Cistus*, *Tuberaria* and *Helianthemum*.

The distribution areas of linages II and VI seem to be mutually exclusive, actually matching different biogeographical provinces of Paleartic realm as delimited by Urdvardy [45]. This suggests that host plants or edapho-climatic conditions play an important role in the distribution of *Picoa* [46,47,48,49,50] and could be driving the speciation process in these hypogeous fungi. However, the existence of additional minor lineages suggests that some degree of sympatric evolution can take place, and so putative hosts are here regarded as the most probable factor behind the observed pattern. Both groups of *Helianthemum* hosts belong in fact to distinct sections and phylogenetic lineages within this genus [51,52]. Unfortunately, the taxonomic treatment of the different ecological lineages in *Picoa* is not fully clear, due to the lack of information about putative *Helianthemum* hosts in the original protologues of *P*. *juniperi* or *Ph*. *lefebvrei* which require more studies. One of the features mentioned by Vittadini in his description of *P*. *juniperi* is that this species was collected under *Juniperus* sp. Only one sample analyzed in the present study matched this condition (AH 39001, Is Arenas, Sardinia, Italy), and its molecular profile linked it to Lineage VI. Interestingly, another feature mentioned by Vittadini was that *P*. *juniperi* specimens fruit in autumn and winter along with other edible truffles, and they are even sold mixed with them in the markets. In the present work, only two samples AH 38893 and AH 39139 (both in Lineage VI) were collected in autumn or early winter (November-December), all others being found in late winter or spring (February-May), excepting samples in Lineage IV, which were all collected even later, in summer (June-July). It is hence tempting to associate the name *P*. *juniperi* with lineage VI (which is also the most widespread in Europe). On the other hand, *P*. *lefebvrei* type was collected at Ras-el-Oued, south of Gabes [13], where the dominant *Helianthemum* host is *H*. *lippii*, a host with uncertain presence in Europe [53]. Because of this host distinction, this name can only be applied to Lineages II or V, and it is tempting to use it for Lineage II, the most widespread in Africa and Middle East. Unfortunately, we lack conclusive ecological information about the putative hosts and fruiting season of the minor lineages (I, III, IV and V), and so we cannot reject the possibility that either *P*. *juniperi* or *P*. *lefebvrei* were proposed for specimens of these clades. It is even possible that some or all subclades within lineage VI (VI-1 to VI-9) should be considered independent taxa if no intermediate specimens can be found and some apomorphic features can be identified (e.g. affinity for a specific *Helianthemum* host). Further studies including richer sampling and providing more accurate data on these key ecological parameters are needed to understand the processes of speciation in these conspicuous species.

## Supporting Information

S1 Text Fungal samples used in this study. (PDF)
