# Supplementary material for: Contrasted Genetic Diversity, Relevance of Climate and Host Plants, and Comments on the Taxonomic Problems of the Genus Picoa (Pyronemataceae, Pezizales)
Source: PLoS One. 2015 Sep 21;10(9):e0138513. doi: 10.1371/journal.pone.0138513 (PMC4577085; doi:10.1371/journal.pone.0138513)
Supplement: S1 Text — (PDF) [file pone.0138513.s001.pdf]

S1 Text. Fungal samples used in this study

Fungal Samples.— ***Picoa* sp**.: **ALGERIA**: Tiaret, Benhamed (Central Algerian Steppe), under *Helianthemum hirtum*, in stand dominated by *Evax pygmaea* and *Scorzonera undulata*, in sandy loam, basic soil, Feb-2009, (BMBH1- BMBH9). Tiaret, Bouchouat (Central Algerian Steppe), under *Helianthemum hirtum* and *H. salicifolium*, in stand dominated by *Artemisia herba-alba*, *Stipa barbata*, in sandy loam, basic soil, Mar-2013, (BMBC10- BMBC31). Tiaret, Sidi Bou Zebboudj (Central Algerian Steppe), under *Helianthemum hirtum*, Mar-2015 (BMBZ32). El-Bayadh, Mesbah (High Steppic Plains) under *Helianthemum* sp., Apr-2015 (BMBD33, BMBD34). Bechar, Beni Ounif (Northwestern Algerian Sahara), under *H. sessiliflorum*, in stand dominated by *Aristida plumosa* and *Retama retam*, in Sandy, basic soil, Mar-2012 (BMBO18- BMBO24). Bechar, Beni Ounif (Northwestern Algerian Sahara), under *H. sessiliflorum*, in stand dominated by *Anvillea radiata* and *Anabasis aretioides*, in Sandy, basic soil, Mar-2012 (BMBT25- BMBT30). **TUNISIA**: Mahdia (Centre East) under *Helianthemum lippii*, in sandy soil, April-2008 (IRA-MBA SBc). Medenine (South East) under *Helianthemum lippii*, in sandy soil, Mar-2015 (IRA-MBA SBa). Sbitla (Center)) under *Helianthemum lippii*, in sandy soil, Mar-2005 (IRA-MBA SBb). **FRANCE**: Marseille, leg. J. Astier, (AH 39205). **GREECE**: Schinias Attica, under *Helianthemum* sp. in forest with *Pinus halepensis* Mill., *Cistus monspeliensis* L., *Pinus pinea* L., *Quercus coccifera* L. and *Pistacia lentiscus* L., in sandy soil, leg. V. Kaounas, 2-II-2011, (VK2043). Ibidem, 12-IV-2011, (VK2148). Rafina Attica, under *Fumana* sp. in forest with *P. halepensis* and *C. monspeliensis*, leg. V. Kaounas, 15-III-2011, (VK2106). **IRAN**: Fars, leg. S. Jamali, 27-III-2011 to 5-IV-2011, (AH 39286, AH 39287). **ISRAEL**: Botsuvha, under *Helianthemum kahiricum* Delile, leg. C. Giovannetti, 2-II-1995, (AH 19584). Botsvuara, under *Helianthemum* sp., leg. C. Giovannetti, 8-II-1995, (AH 39204).

ITALY: L'Aquila, Lucoli, 27-VI-1982, (AH 39285). Oristano, Is Arenas, in a desertic area, leg. P. Fantini, 2-V-1999, (AH 39207). Ibidem, 3-V-1999, (AH 39206). Oristano, Is Arenas, close to *Juniperus oxycedrus*, *Cistus* sp., *Ephedra* sp. and *Pistacia* sp., leg., A. Montecchi, 3-V-1999, (AH 39001). Oristano, Is Arenas- S. Vero Milis, under *Cistus* sp., *Quercus* sp. and *Helianthemum* sp., leg. L. Gori, 1-V-1999, (AH 39282). **SPAIN**: Albacete, Casas de Lázaro, leg. A. Rodríguez, 14-V-2004, (AH 39035). Burgos, Milagros, under *Quercus ilex* L. and *Quercus faginea* Lam., leg. N. Redondo, 10-VII-2002, (AH 38931). Burgos, Peñalara de Aranda, in steppe, leg. N. Redondo, 28-V-2007, (AH 38906). Burgos, Llanos de la Bureba, sandy soil with *Cistus salviifolius* L., *Tuberaria guttata* (L.) Fourr. and *Helianthemum* sp., leg. Francisco Sáinz, 19-V-2007, (AH 39246). Burgos, Cornudilla, road with *Pinus* sp. and *Q. ilex*, leg. Francisco Sáinz, 20-VI-2010, (AH 39247). Burgos, Rojas de Bureba, country road, leg. Francisco Sáinz, 3-V-2008, (AH 39248). Ciudad-Real, Malagon, under *Helianthemum ledifolium* Mill. and *Helianthemum salicifolium* (L.) Mill., leg. M. Bastante, 21-IV-2002, (AH 37802). Córdoba, Priego de Córdoba, under *Q. ilex*, leg. J. Gómez, IV-1991, (AH 19561). Madrid, Aranjuez, hills oposite to 'Casita del Príncipe', gypsicolous soil with *Helianthemum* sp., leg. G. Moreno, F. Prieto, J. Diez, H. Singer, N. Ayala, 7-IV-2002, (AH 37794). Madrid, Alcalá de Henares, Cerro 'El Viso', basic soil with *Helianthemum* sp., leg. M.A. Sanz, P. Alvarado, J.L. Manjón, 13-IV-2011, (AH 39268, AH 39269, AH 39270). Madrid, Colmenar de Oreja, Valdeguerra, leg. G. Moreno, J. Cámara, H. Kreisel, K. Kreisel, 18-IV-1999, (AH 38913). Guadalajara, Castilblanco de Henares, calcareous soil with *Thymus* sp, leg. M.A. Sánz, 26-XII-2009, (AH 38893). Ibidem, 29-V-2010, (AH 38956). Guadalajara, Membrillera, under *Helianthemum* sp., leg. M.A. Sánz, 6-XI-2010, (AH 39139). Zaragoza, Bujaraloz, leg. M. Obregón, 21-III-2004, (AH 37801, AH 38914).
